# Chromosome-scale genome assembly of sweet tea (*Lithocarpus polystachyus* Rehder)

**DOI:** 10.1038/s41597-023-02791-y

**Published:** 2023-12-06

**Authors:** Hui Liu, Rengang Zhang, Biao-Feng Zhou, Zhao Shen, Xue-Yan Chen, Jie Gao, Baosheng Wang

**Affiliations:** 1 State Key Laboratory of Plant Diversity and Specialty Crops/Guangdong Provincial Key Laboratory of Applied Botany, South China Botanical Garden, Chinese Academy of Sciences, Guangzhou, 510650 Guangdong China; 2 South China National Botanical Garden, Chinese Academy of Sciences (CAS), Guangzhou, China; 3 Yunnan Key Laboratory for Integrative Conservation of Plant Species with Extremely Small Populations/Key Laboratory for Plant Diversity and Biogeography of East Asia, Kunming Institute of Botany, Chinese Academy of Sciences, Kunming, 650201 Yunnan China; 4 CAS Key Laboratory of Tropical Forest Ecology, Xishuangbanna Tropical Botanical Garden/Center of Conservation Biology, Core Botanical Gardens, Chinese Academy of Sciences, Menglun, 666303 Yunnan China

**Keywords:** Genomics, Gene expression

## Abstract

*Lithocarpus*, with >320 species, is the second largest genus of Fagaceae. However, the lack of a reference genome limits the molecular biology and functional study of *Lithocarpus* species. Here, we report the chromosome-scale genome assembly of sweet tea (*Lithocarpus polystachyus* Rehder), the first *Lithocarpus* species to be sequenced to date. Sweet tea has a 952-Mb genome, with a 21.4-Mb contig N50 value and 98.6% complete BUSCO score. In addition, the per-base consensus accuracy and completeness of the genome were estimated at 60.6 and 81.4, respectively. Genome annotation predicted 37,396 protein-coding genes, with repetitive sequences accounting for 64.2% of the genome. The genome did not undergo whole-genome duplication after the gamma (γ) hexaploidy event. Phylogenetic analysis showed that sweet tea diverged from the genus *Quercus* approximately at 59 million years ago. The high-quality genome assembly and gene annotation resources enrich the genomics of sweet tea, and will facilitate functional genomic studies in sweet tea and other Fagaceae species.

## Background & Summary

*Lithocarpus* is the second largest genus of the Fagaceae family, comprising more than 320 species^1^. Species belonging to the genus *Lithocarpus*, commonly known as stone oaks, are dominant canopy trees in the tropical and subtropical forests of East Asia^2^. *Lithocarpus polystachyus* Rehder (2n = 2x = 24; syn. *Lithocarpus litseifolius* [Hance] Chun) is an evergreen plant widely distributed in south China and the adjacent southeast Asian countries^3^. The *L. polystachyus* is commonly known as “sweet tea”, because its leaves have a sweet taste when brewed. The leaves of *L. polystachyus* also have medicinal properties and have long been used as herbal tea to prevent and manage diabetes.

The leaves of *L. polystachyus* contain high concentration of dihydrochalcones (DHCs), which is the main source of its sweet taste. DHCs is a class of minor flavonoids (e.g. trilobatin and neohesperidin), which have been reported to act as flavor sweeteners and bitterness blockers^4–6^. DHCs such as phloretin, phlorizin, and sieboldin have also been demonstrated to play important roles in human health by providing a wide range of beneficial effects against diabetes, cardiovascular, cancer, and free radical-involving diseases^7–10^. In addition, phloretin exhibits strong broad-range bactericidal and fungicidal activities, and sieboldin is a powerful multipotent antioxidant^9,11^. DHCs have been isolated from many medicinal plants belonging to different families, but their contents vary significantly both among and within species^12–15^. Four DHCs (phloretin, phlorizin, trilobatin, and sieboldin) have been reported in sweet tea, and the biosynthesis pathway of the first three DHCs have been proposed^14,16^. However, our knowledge of DHC biosynthesis and regulatory mechanisms is limited in sweet tea. Specifically, candidate genes and transcription factors involved in the DHCs biosynthesis pathway remain to be investigated.

Here, we assembled a high-quality chromosome-scale genome of sweet tea, the first ever in the genus *Lithocarpus*, using PacBio HiFi and Hi-C data. The assembled sweet tea genome had a total length of 952 Mb, with a contig N50 of 21.4 Mb and a complete BUSCO score of 98.6%. A total of 922.6 Mb (96.9%) of the sequences were anchored to the 12 chromosomes. Genome annotation predicted 37,396 protein-coding genes and 597.5 Mb repetitive sequences. The high-quality sweet tea genome provides a valuable resource for exploring key genes and molecular regulatory mechanisms involved in the biosynthesis of important compounds, including DHCs, and will further serve as a strong foundation for trait improvement in this species.

## Methods

### Plant material and sequencing

**Fig. 1:**
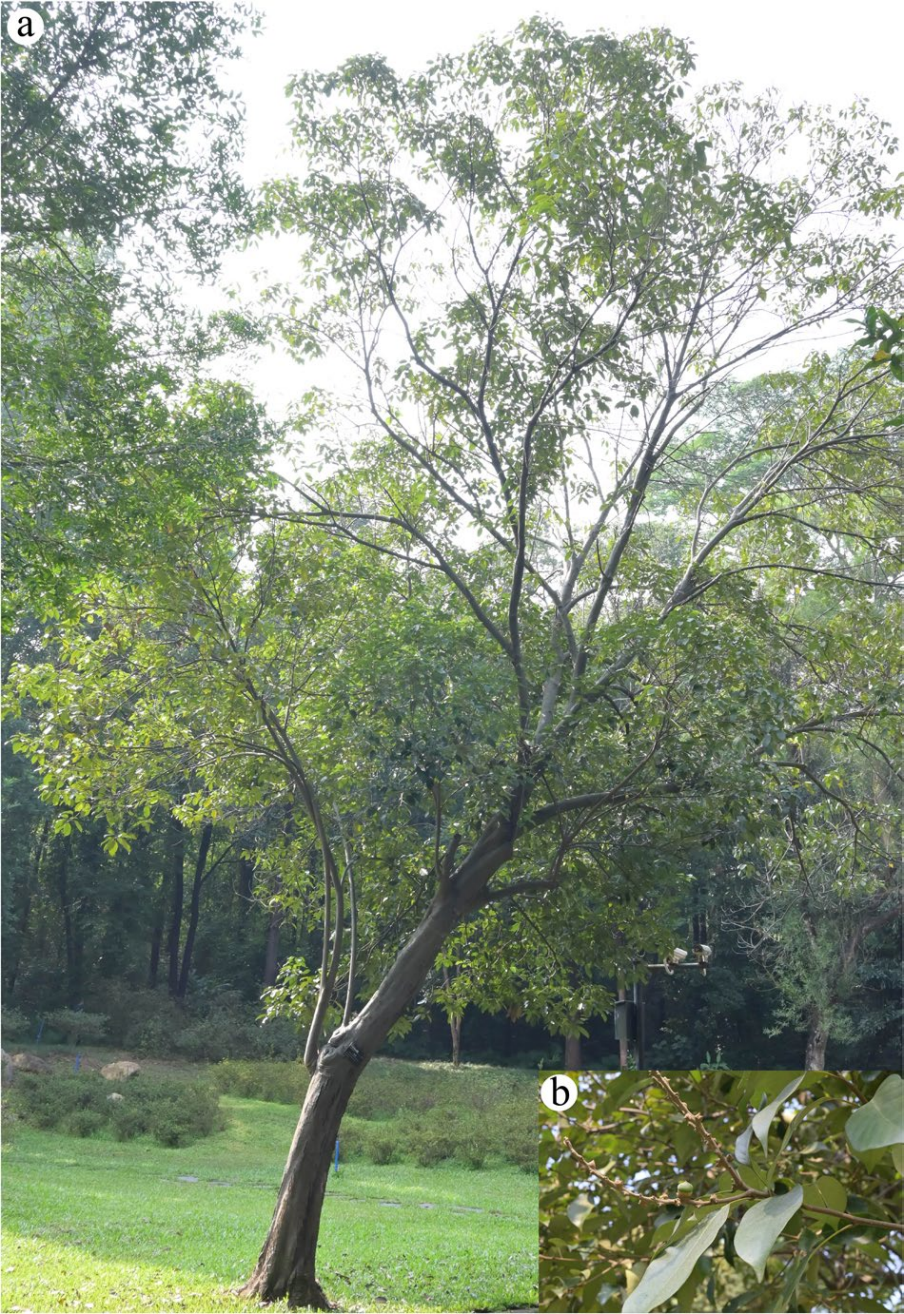
The sweet tea tree sequenced in this study. (**a**) The sequenced tree planted in South China National Botanical Garden (accession number: 19940074) (**b**) The fruits of sequenced tree.

A healthy sweet tea tree growing in the South China National Botanical Garden (accession number: 19940074) was selected for *de novo* genome assembly (Fig. 1). Young leaves were collected from the selected individual for whole-genome sequencing. The leave, stem, and root were collected for RNA-sequencing (RNA-seq) for the transcriptome assembly. All samples were immediately flash-frozen in liquid nitrogen after harvest, and stored at −80 °C for subsequent nucleic acid extraction.

Genomic DNA and total RNA were isolated from the leaves using DNeasy Plant MiniKit (Qiagen, Germany) and RNAprep Pure Plus Kit (Tiangen, China), respectively. The mRNA was purified from total RNA using poly-T oligo-attached magnetic beads for subsequent sequencing. To perform short-read sequencing for genome survey, transcriptome assembly, and transcriptomic profiling, libraries with an insert size of ~350 bp was constructed and sequenced on the Illumina NovaSeq 6000 platform to generate 150-bp paired-end reads. To perform *de novo* genome assembly, a 15–20-kb PacBio HiFi library was prepared using the SMRTbell Express Template Preparation Kit 2.0 (Pacific Biosciences, USA) and sequenced on the PacBio Sequel IIe platform to produce PacBio HiFi long reads. To generate the high-throughput chromatin conformation capture (Hi-C) data, DNA was isolated from the leaves and fixed with paraformaldehyde. The genomic DNA was then enzymatically digested with *DnpII*, generating fragments with sticky ends. These sticky ends were repaired by ‘A’ or ‘C’ deoxynucleotides with biotin by using DNA polymerase. Subsequently, the DNA fragments were ligated together to form chimeric circles using DNA ligase. The ligated DNAs were then uncrosslinked, purified, and sheared into 300–500 bp in size. Finally, the Hi-C sequencing library was sequenced on the Illumina NovaSeq 6000 platform, generating 150-bp paired-end reads.

A total of 32.8 Gb PacBio HiFi long reads (~37.6 × coverage), 118.8 Gb Hi-C reads (~135.9 × coverage), 44.3 Gb (~51 × coverage) paired-end Illumina reads, and 21.5 Gb RNAseq reads were generated for the genome assembly, genome survey, and transcriptome assembly.

### Genome survey

**Fig. 2:**
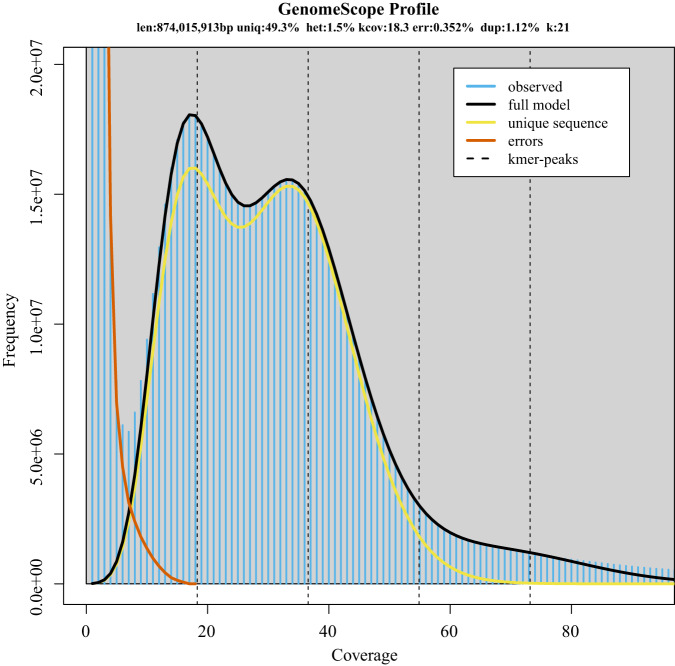
*K*-mer (21-mer)-based estimation of genome characters of sweet tea.

The 21-bp *K*-mers with Illumina reads were counted using Jellyfish v1.1.11^17^, with default parameters. The genome size, the level of heterozygosity, and repeat content were estimated using GenomeScope v1.0^18^. The estimated genome was 874 Mb in length and a heterozygosity of 1.5% (Fig. 2).

### *De novo* genome assembly

The HiFi long reads were initially assembled into contigs using hifiasm v0.18.6-r513^19^ with default parameter to generate one primary haplotype-collapsed assembly and a pair of partially phased assemblies. The primary assembly was selected for further scaffolding because it was much more contiguous than the partially phased assemblies. To anchor the contigs onto scaffolds, the Hi-C reads were mapped on to the contigs using Juicer v2.0^20^ with *Dpn*II (GATC) as the restriction enzyme site, and scaffolds were generated using the 3D-DNA (v201008) pipeline^21^ with the following parameters: “-m haploid -i 150000 -r 0--editor-repeat-coverage 5”. Based on the chromosome number of sweet tea (n = 12) determined previously^22,23^ and the interaction information of Hi-C reads generated with the 3D-DNA pipeline, the chromosome segmentation boundaries and assembly errors were manually checked and adjusted using Juicebox v1.11.08^24^.

**Fig. 3:**
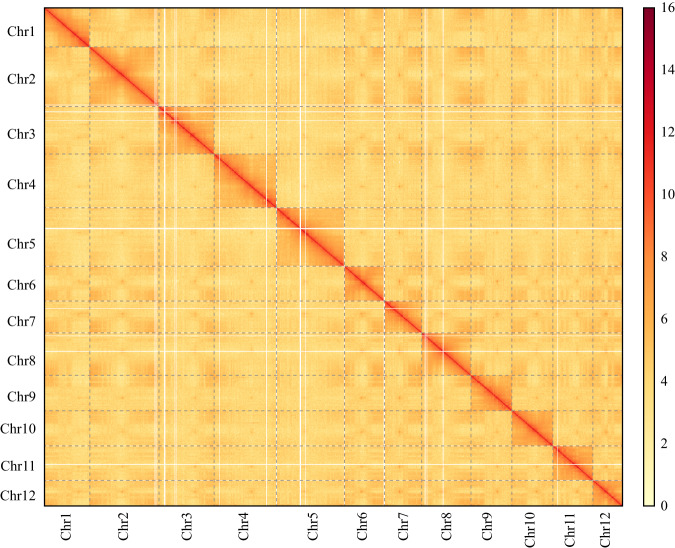
Genome-wide chromatin interaction heatmap of sweet tea based on Hi-C data.

The total length of the final sweet tea genome assembly was 952.3 Mb, which is slightly larger than the genome size estimated by *K*-mer analysis (Fig. 2 and Table 1), and smaller than the size measured by flow cytometry (ca. 1,149 Mb)^22^. The contig and scaffold N50 values of the sweet tea genome were 21.4 and 78.6 Mb, respectively, which are comparable with those of recently published Fagaceae genome assemblies (Table 2). A total of 922.6 Mb (96.9%) of the sequences were anchored to the 12 chromosomes (Table 1). The Hi-C interaction map showed a strong intrachromosomal interactive signal along the diagonal (Fig. 3).

**Table 1:** Statistics of the sweet tea genome assembly and annotation.

**Genome assembly**	
Total assembly size (Mb)	952.3
GC content (%)	35.64
Number of scaffolds	521
Maximum contig length (Mb)	61.6
Contig N50 (Mb)	21.4
Number of gaps	60
Anchor rate (%)	96.9
BUSCO (%)	98.6
LAI	21.5
QV	60.6
K-mer completeness	81.4
Mapped HiFi reads (%)	99.9
**Genome annotation**	
Repetitive sequences (%)	64.2
Number of protein-coding genes	37,396
Average gene length (bp)	4,903
Average CDS length (bp)	1,163
Average intron length (bp)	948
BUSCO (%)	97.1
**Functional annotation**	
Swiss-Prot	28,087 (75.1%)
TrEMBL	35648 (95.5%)
Nr	35,987 (96.2%)
InterPro	33,072 (88.4%)
eggNOG	34,072 (91.1%)
KEGG	27,396 (73.3%)
GO	26,220 (70.1%)
Total	36,096 (96.5%)

**Table 2:** Summary of the genomic features of 12 Fagaceae species.

Species	Size (Mb)	Scaffold N50 (Mb)	Contig N50 (Mb)	Gene number	Average gene length (bp)	Average intron length (bp)
Sweet tea	952	78.6	21.4	37,396	4,903	948
*Quercus dentata*^65^	894	75.6	4.2	31,584	5,243	709
*Quercus lobata*^66^	844	66.4	1.0	36,703	5,169	859
*Quercus mongolica*^67^	810	66.7	2.6	36,553	6,085	1,253
*Quercus variabilis*^68^	788	64.9	64.9	36,756	5097	962
*Quercus acutissima*^69^	758	63.3	1.4	30,623	5,601	1,134
*Quercus gilva*^70^	890	70.4	28.3	36,442	3,724	761
*Castanea crenata*^71^	718	61.8	6.4	41,399	3,539	877
*Castanea mollissima*^72^	774	65.8	5.9	45,011	3,330	790
*Castanopsis hystrix*^73^	883	75.6	41.0	37,750	5,072	1,117
*Castanopsis tibetana*^74^	879	76.7	3.3	40,937	4,857	1,120
*Fagus sylvatica*^75^	541	46.6	0.1	63,736	3,942	391

### Identification and characterization of repetitive elements

Tandem duplications were identified using TRF 4.09^25^, and both structurally intact and fragmented transposable elements (TEs) were annotated using EDTA (Extensive *de-novo* TE Annotator) v1.9.6^26^. Overlapping regions of each class of repetitive elements were counted only once when calculating their total size. The divergence (*K*) of intact LTRs identified was estimated by Kimura two-parameter distance (K2P)^27^. The insertion time was calculated by the formula: *T* = *K*/(2 × *r*), where *r* refers to a substitution rate of 1.3 × 10^–8^ per site per year^28^.

**Fig. 4:**
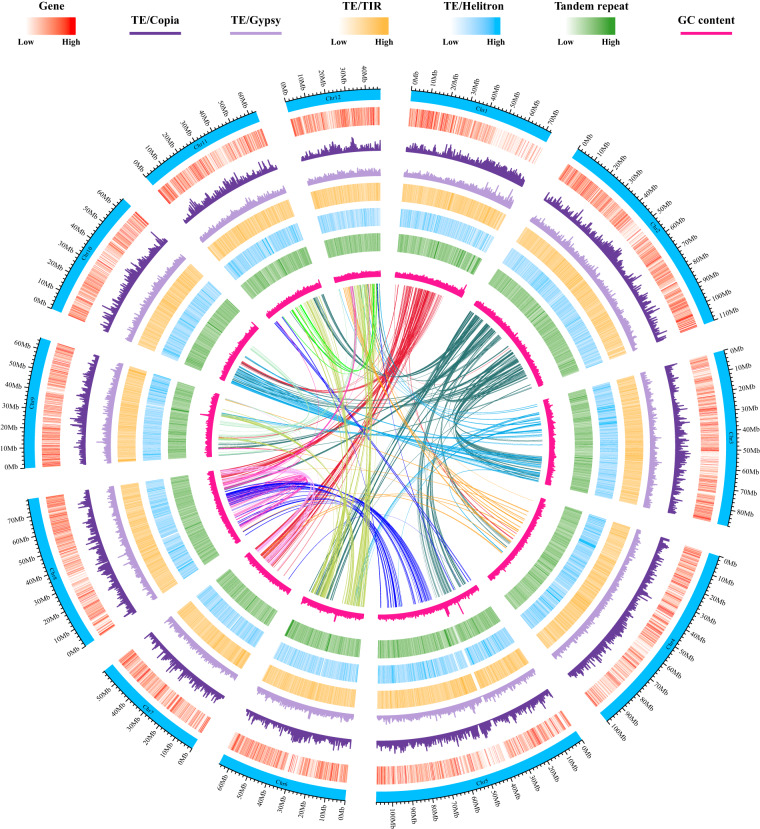
Genomic features of sweet tea. The tracks from the outer to the inner circle represent the 12 chromosomes (Chr1–Chr12), gene density, trnasposable element (TE; Copia, Gypsy, TIR, and Helitron) density, tandem repeat density, and GC content. The connecting lines in the center of the Circos plot indicate the syntenic gene blocks.

**Fig. 5:**
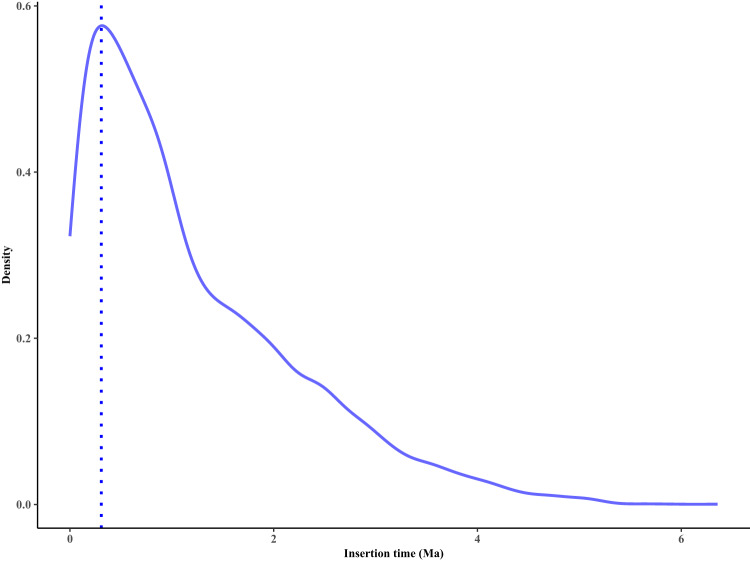
The distribution of insertion time of intact LTRs in sweet tea. Ma, million years ago.

A total of 597.5 Mb (62.74%) of the assembled sequences were annotated as TEs, with LTR (33.49%), TIR (14.27%), and Helitron (10.82%) being the three most abundant TE superfamilies (Fig. 4 and Table 3). We found most of the LTRs have been accumulated recently over a short time span with the peak of 0.3 million years ago (Ma), suggesting an expansion event (Fig. 5). In addition, TEs were unevenly distributed along the genome, with greater accumulation in regions with low gene density (Fig. 4).

**Table 3:** Summary of the repetitive sequences in the sweet tea genome assembly.

Class	Count	Length (bp)	Percent (%)
Retrotransposon	411,856	319,789,051	33.58
LTR	409,558	318,946,298	33.49
Copia	134,834	110,702,254	11.62
Gypsy	139,857	144,704,196	15.19
Others	134,867	69,696,151	7.32
Non-LTR	2,298	843,058	0.09
LINE	2,267	836,912	0.09
Others	31	6,146	0
DNA	734,444	225,638,194	23.69
TIR	422,024	135,888,531	14.27
CACTA	86,286	28,213,102	2.96
PIF/Harbinger	73,142	21,304,551	2.24
Tc1/Mariner	13,178	4,376,889	0.46
Mutator	168,896	51,025,541	5.36
hAT	80,522	36,908,975	3.88
Helitron	312,391	103,077,557	10.82
Polintons	29	16,840	0
Unclassified TE	194,523	70,412,435	7.39
Total TE	1,340,823	597,528,333	62.74
Tandem repeat	565,919	60,218,687	6.32
Total repetitive sequences	1,906,742	611,661,987	64.23

### Gene prediction and functional annotation

Protein-coding genes in the sweet tea repeat-masked genome were predicted by applying MAKER v3.01.04^29^ to the combined results of RNA-seq-based prediction, protein-homology-based prediction, and *ab initio* prediction. Trinity v2.14.0^30^ was used to *de novo* assemble the transcriptome for RNA-seq-based gene prediction. HiSat2 v2.2.1^31^ was used to align the RNA-seq reads against the sweet tea genome, and then Trinity and StringTie v2.2.1^32^ were used to assemble the genome-guided transcriptomes. Then, the *de novo* and genome-guided transcriptome assemblies were merged and refined using CD-HIT-EST v4.8.1^33^, generating the transcript-based gene prediction. Plant protein sequences from the Swiss-Prot database (https://www.uniprot.org/downloads) and annotated proteins from *Arabidopsis thaliana* Araport11^34^ and *Populus trichocarpa* v4.1^35^ were used for protein-homology-based prediction. AUGUSTUS v3.4.0^36,37^ with BUSCO single-copy genes as training data, GeneMark-ES v4.69_lic^38^ with a self-training algorithm, and SNAP v2006-07-28^39^ with the output of MAKER as training data were used for *ab initio* prediction. MAKER was run for a total of three rounds to obtain high-quality gene models.

To further improve the gene prediction, genes predicted by MAKER were integrated into consensus gene models using EVidenceModeler v1.1.1^40^ and then further polished using PASA v2.5.2^41^. The longest transcript of each predicated gene model was considered as the representative gene models. The completeness of gene models was assessed by searching the gene content of the embryophyta_odb10 database using BUSCO v5.4.3^42^.

Functional annotations were performed using different tools: InterProScan v5.56-89.0^43^ for predicting potential protein domains; KofamKOALA v1.3.0^44^ for determining KEGG ortholog assignments; and eggNOG-mapper v2.1.9^45^ for inferring orthology assignments. In addition, BLASTP v2.13.0 + was used to search the homologous sequences in Nr, Swiss-Prot, and TrEMBL databases. The GO (Gene ontology) terms of the genes were extracted from the eggNOG and InterPro entries. Transcription factors were annotated using PlantTFDB v5.0^46^ and PlanTFcat^47^.

A total of 37,396 protein-coding genes were predicted, with 97.1% of the complete BUSCO genes covered (Table 1). The average length of genic regions, coding sequences (CDSs), and intron sequences was 4,903 bp, 1,163 bp, and 948 bp, respectively. The average length of genes and introns in sweat tea was comparable to that of Fagaceae species (Table 2). Among the predicted protein-coding genes, 36,096 (96.5%) were annotated in functional databases (Tables 1), and 3,200 were identified as transcription factor (TF) genes belonging to 99 different families.

### Comparative genomic analysis

To investigate the syntenic relationships between the protein-coding genes of sweet tea and those of the other Fagaceae species, collinear blocks between sweet tea and the representative species of each genus or section were identified based on protein sequences using MCScan implemented in jcvi v1.2.7^48^. The synonymous substitution rate (Ks) was estimated using KaKs_Calculator v3.0^49^ based on paralogous gene pairs extracted from the collinear blocks.

**Fig. 6:**
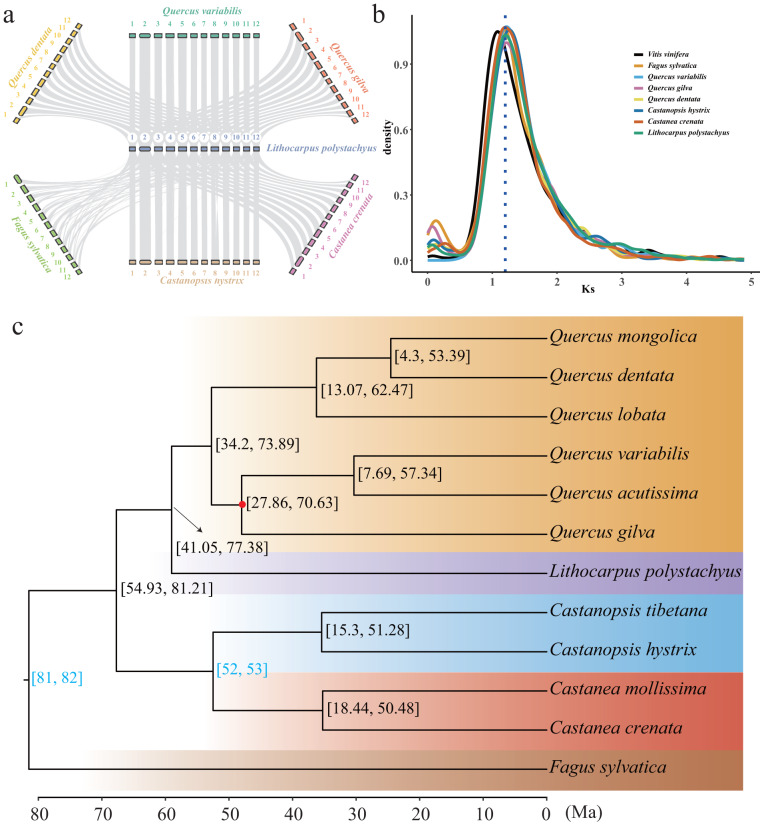
Syntenic anasis between sweet tea and other Fagaceae species. (**a**) Syntenic blocks between sweet tea and other Fagaceae species. (**b**) Distribution of the synonymous substitution (Ks) rates of paralogous gene pairs within syntenic blocks. The blue dashed line represents the 1.2 of Ks. (**c**) Phylogenetic tree of sweet tea and 11 other Fagaceae species. The numbers in square brackets indicate the 95% confidence intervals of the divergence time, and two fossil calibrations are indicated in blue. The red solid circle represents support lower than 100%, based on 1,000 bootstrap replicates.

The syntenic gene blocks showed 1:1 syntenic patterns between sweet tea and other Fagaceae species (Fig. 6a), suggesting a conserved genome structure in Fagaceae. The Ks distribution pattern of the sweet tea syntenic genes presented the same signature of Ks peaks only at ~1.2 as that observed in the other Fagaceae and *Vitis vinifera* genomes, indicating that the genome of sweet tea did not undergo WGD after the gamma (γ) hexaploidy event (Fig. 6b).

### Phylogenetic analyses

We conducted phylogenetic analyses to infer the relationship between sweet tea and other Fagaceae species. To do that, we utilized OrthoFinder v2.5.4^50^ to identify orthologous genes between 12 Fagaceae species (Table 2). A total of 3,498 single-copy orthologous genes identified by OrthoFinder were aligned using MAFFT v7.505^51^, trimmed using trimAl v1.4.rev15^52^, and then concatenated together. Based on the concatenated alignment, a maximum likelihood phylogenetic tree was constructed using IQ-TREE v2.0.5^53,54^. The divergence times among Fagaceae species were estimated using MCMCTree in the PAML v4.9j package^55^, based on four-fold degenerate sites. Two fossil calibrations were used to constrain the age of nodes: (1) the split between the *Fagus* genus and the rest of the Fagaceae species at 82–81 Ma^56^, and (2) the divergence between the *Castanopsis* and *Castanea* genera at 53–52 Ma^57^. The phylogenetic analyses showed that sweet tea was sister to the genus *Quercus*, with strong bootstrap support (100%). Calibration of the phylogenetic tree showed that sweet tea diverged from the genus *Quercus* ~59 Ma (95% HPD: 41.05–77.38 Ma) (Fig. 6c).

## Data Records

The genome assembly have been deposited in the GenBank database of NCBI with accession number JAWTZU000000000^58^. The raw sequence data have been deposited in the Genome Sequence Archive (GSA) in National Genomics Data Center (NGDC) database (https://ngdc.cncb.ac.cn/) under the accession number CRA012397^59^. The genome annotation files and synteny data were deposited in the Figshare database^60^.

## Technical Validation

Genome completeness was assessed by searching the gene content of the embryophyta_odb10 database with Benchmarking Universal Single-Copy Orthologs (BUSCO) v5.4.3^42^; the quality of repetitive genomic regions was assessed using the LAI vbeta3.2 program (Ou *et al*., 2018); per-base consensus accuracy (QV) and k-mer completeness was estimated with Merqury v1.3^61^ using PacBio HiFi long reads with a *K*-mer value of 20-bp; and PacBio HiFi long reads were mapped on to the genome using minimap2 v2.24-r1122^62^ to calculate the mapping rate. The telomeric sequences in the sweet tea genome assembly were identified using quartet v1.1.3^63^ with “-c plant”.

**Fig. 7:**
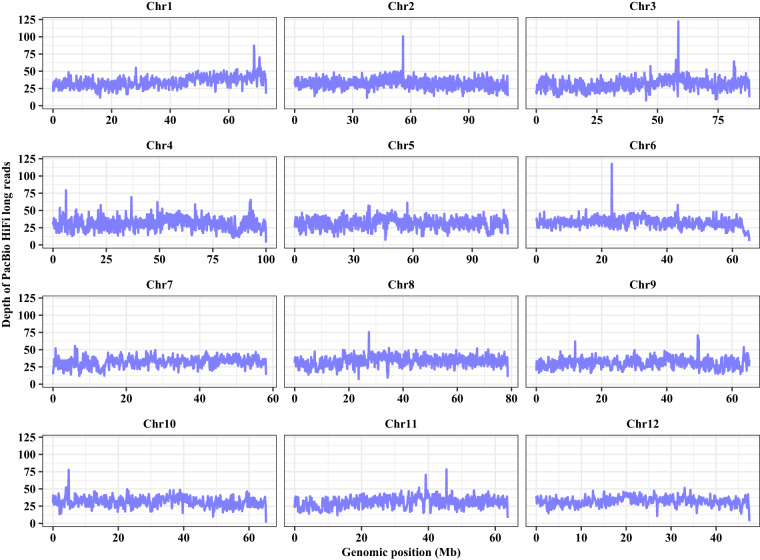
Coverage of HiFi long reads mapped across the 12 chromosomes of sweet tea.

The sweet tea genome assembly showed a high degree of completeness and accuracy at the chromosome scale as indicated by the following statistics (Table 1): (1) the complete BUSCO score was 98.6%, which suggests high-gene space completeness of the assembly; (2) the LTR Assembly Index (LAI) was 21.5 for the assembly, which suggested it was a high quality genome^64^; (3) the values of QV and k-mer completeness were estimated as 60.6 and 81.4, respectively, based on the analysis of 20-mer spectra from the PacBio HiFi long reads; (4) 99.88% of PacBio HiFi long reads were aligned to the sweet tea genome assembly, with an average coverage depth of ~32 × along the 12 chromosomes (Fig. 7); and (5) telomeric sequences were detected at both ends of two chromosomes (Chr5 and Chr10).

## Data Availability

No custom code was used for this study. All data analyses were conducted using published bioinformatics software with default settings, unless otherwise specified.
